# Immunization with *L. sigmodontis* Microfilariae Reduces Peripheral Microfilaraemia after Challenge Infection by Inhibition of Filarial Embryogenesis

**DOI:** 10.1371/journal.pntd.0001558

**Published:** 2012-03-06

**Authors:** Sebastian Ziewer, Marc P. Hübner, Bettina Dubben, Wolfgang H. Hoffmann, Odile Bain, Coralie Martin, Achim Hoerauf, Sabine Specht

**Affiliations:** 1 Institute of Medical Microbiology, Immunology and Parasitology, University Hospital Bonn, Bonn, Germany; 2 Institute of Tropical Medicine, University Hospital Tuebingen, Tuebingen, Germany; 3 UMR 7245 MCAM MNHN CNRS, Muséum National d'Histoire Naturelle, Paris, France; McGill University, Canada

## Abstract

**Background:**

Lymphatic filariasis and onchocerciasis are two chronic diseases mediated by parasitic filarial worms causing long term disability and massive socioeconomic problems. Filariae are transmitted by blood-feeding mosquitoes that take up the first stage larvae from an infected host and deliver it after maturation into infective stage to a new host. After closure of vector control programs, disease control relies mainly on mass drug administration with drugs that are primarily effective against first stage larvae and require many years of annual/biannual administration. Therefore, there is an urgent need for alternative treatment ways, i.e. other effective drugs or vaccines.

**Methodology/Principal Findings:**

Using the *Litomosoides sigmodontis* murine model of filariasis we demonstrate that immunization with microfilariae together with the adjuvant alum prevents mice from developing high microfilaraemia after challenge infection. Immunization achieved 70% to 100% protection in the peripheral blood and in the pleural space and furthermore strongly reduced the microfilarial load in mice that remained microfilaraemic. Protection was associated with the impairment of intrauterine filarial embryogenesis and with local and systemic microfilarial-specific host IgG, as well as IFN-γ secretion by host cells from the site of infection. Furthermore immunization significantly reduced adult worm burden.

**Conclusions/Significance:**

Our results present a tool to understand the immunological basis of vaccine induced protection in order to develop a microfilariae-based vaccine that reduces adult worm burden and prevents microfilaraemia, a powerful weapon to stop transmission of filariasis.

## Author Summary

Lymphatic filariasis is caused by parasitic filarial worms that are transmitted by mosquitoes, requiring uptake of larvae and distribution into the blood of the host. More than 120 million people are infected and about 30% of these individuals suffer from clinical symptoms. Reduction in transmission currently depends on mass drug administration, which has significantly reduced transmission rates over the past years. However, despite repetitive rounds of administration, transmission has not been eliminated completely from endemic areas. In some infected individuals the immune system can partially control the parasite, such that a proportion of infected individuals remain microfilaria-negative, despite the presence of adult worms. Therefore mechanisms must exist that are able to combat microfilaraemia. Identifying such mechanisms would help to design vaccines against disease transmitting microfilarial stages. Using the *Litomosoides sigmodontis* murine model of filariasis research we show a successful immunization against the blood-circulating larval stage that is responsible for arthropod-dependent transmission of the disease. Reduced microfilaraemia was associated with impairment of worm embryogenesis, with systemic and local microfilarial-specific host IgG and with IFN-γ secretion by host cells at the site of infection. These results raise hope for developing a microfilariae-based vaccine, being a pivotal step towards eradicating filariasis.

## Introduction

Infections with filarial nematodes are classified among the “neglected tropical diseases” and cause serious public health problems in the tropics and subtropics with more than 150 million people infected and many more at risk. Lymphatic filariasis (LF) caused by the filarial nematodes *Wuchereria bancrofti* and *Brugia* spp. affects 120 million people with one third of them suffering from clinical presentations of the infection, namely lymphedema of the extremities and hydrocele, making LF the second-largest cause of long-term disability [1].

Human filariasis is transmitted by blood feeding vectors that ingest first stage larvae (microfilariae, Mf) from infected patients. Within the vector, Mf undergo two obligatory molts to become infective third stage larvae (L3). After their transmission to a new host infectious L3 molt twice into adult worms, which mate and release thousands of new Mf [2].

Current elimination strategies of the WHO such as the Global Programme to Eliminate LF (GPELF [3]) or the African Programme for Onchocerciasis Control (APOC [4]) are based on the mass drug administration (MDA) of the microfilaricides ivermectin (IVM), diethylcarbamazine and albendazole that have been successful in reducing Mf-burden. However, only IVM and albendazole are used in MDA programs against LF in Africa, because diethylcarbamazine causes rapid death of Mf, thereby increasing chances of adverse reactions, such as ocular damage in onchocerciasis [2]. In addition, doxycycline has been introduced for individual drug administration [5] directed against the obligate endosymbiotic *Wolbachia* bacteria of the filariae [6], [7]. Doxycycline inhibits filarial embryogenesis, and has been proven to be macrofilaricidal and to halt or reduce pathology [8], [9]. However, doxycycline is contraindicated in children ≤9 years and pregnant woman and improvement of anti-wolbachial chemotherapy to be used in public health control programs is a focus of ongoing research [2].

Despite the success of anti-helmintic drugs used in MDA programs in order to reduce infection and morbidity, certain drawbacks have to be considered. IVM has only limited macrofilaricidal efficacy [2] and repeated treatment for the life of the adult worm (up to eight years) is needed in order to stop transmission. Together with the limited logistics, especially in areas with civil unrest, the occurrence of adverse events after treatment such as scrotal pain or systemic inflammation can substantially corrupt the degree of compliance to therapy [1]. Finally, emerging resistance to drugs [10] reinforces the urgent need of alternative ways of disease control.

Hence, besides drug therapy and vector control [11], the development of a vaccine against filarial infections would be a pivotal step towards the elimination of this disease [12]. As filarial nematodes have a high reproductive capacity with a total daily turnover of thousands of Mf in chronically infected human individuals [13], a vaccine achieving substantial clearance of circulating Mf would be a step towards stopping disease transmission. At best, a vaccine would be used in conjunction with MDA after Mf loads in a population were reduced either to prevent re-infection or to prevent circulation of Mf in the blood, particularly in areas of very high transmission.

Despite the severity of infection and the vast number of infected people and individuals at risk, there is no vaccine against filarial infections available [12], [14]. Addressing this issue, various animal immunization studies using different approaches have been conducted. For example the group of Odile Bain used *Litomosoides sigmodontis* L3 to immunize mice and achieved up to 58% protection [15]. The protection was established within a few days after challenge infection and was characterized by L3-specific immunoglobulins, eosinophilia and high levels of IL-5. Lange and colleagues used a similar approach to immunize mice with *Onchocerca volvulus* L3 and also observed fast protection, which led to reduction of the recovery rates by 54% to 77% between five and 28 days post challenge infection (p.i.), and this was also associated with eosinophils and IL-5 [16]. In the experiments of Dixit el al [17], immunization of *Mastomys coucha* with a fraction of adult *B. malayi* extract reduced the recovery rate of adult worms by 85.7%. Other groups used recombinant peptides instead of complete extracts [18], [19], [20], [21]. The immunization with *B. malayi* heavy chain myosin for example generated a high level of protection against challenge infection in jirds and *M. coucha* [21]. Different to all these setups, Anand and colleagues used a cocktail of *B. malayi* DNA to immunize mice and found high cytotoxcicity against *B. malayi* Mf in immunized mice, associated with specific Ig and increased IFN-γ responses [22]. However, none of these studies reported complete clearance of adults or Mf.

In the 1960s, Wenk and colleagues found that cotton rats immunized with *L. sigmodontis* Mf had fewer blood-circulating Mf, although adult worms were present [23], [24], [25]. In this study we have taken this approach another step forward using the fully permissive BALB/c mouse model to study filarial infections. In this model with the advantage of greater access to immunological tools, female *L. sigmodontis* worms release the Mf into the pleural space of the thoracic cavity, the site of infection. From there they migrate into the peripheral blood [26]. Here, we show that immunization with Mf together with the adjuvant alum reduces microfilaraemia by apparently inhibiting embryogenesis.

## Materials and Methods

### Animals

Eight - 12 week old female BALB/c wild type mice (Janvier, La Genest St. Isle, France) were maintained under specific pathogen-free conditions, according to animal welfare guidelines.

### Ethics Statement

All animal experiments were approved by and conducted in accordance with guidelines of the appropriate committee (Landesamt für Natur, Umwelt und Verbraucherschutz, Köln, Germany).

### Immunization

Mf were purified from the peripheral blood of infected cotton rats on a Percoll gradient as described [27]. In brief, isoosmotic Percoll (Sigma-Aldrich, Munich, Germany) was prepared by mixing 9 parts of Percoll (density, 1.130 g/ml) with 1 part of 2.5 M D-sucrose (Sigma-Aldrich). Dilutions of the isoosmotic Percoll in 0.25 M sucrose were made to obtain 25% and 30% solutions. Three ml of both gradient dilutions were layered and the peripheral blood diluted 1∶2 in phosphate-buffered saline (PBS) (PAA, Cölbe, Germany) was placed on top. After centrifugation at 400×g for 30 min at room temperature (RT) without brakes, Mf (between the 25% and 30% layers) were recovered and washed twice with PBS and 1×10^5^ viable Mf per mouse were used for each immunization. Injection was performed via different administration routes as indicated in the text (see also Figure S1). For Mf attenuation, 1×10^6^ Mf/ml were irradiated 40 min at 140 kV and 25 mA, corresponding to an absorbed dose of 400 Gray (Gy) at the Facility of Experimental Therapy of the University Hospital Bonn. Microscopic analysis of irradiated Mf confirmed their attenuation by monitoring their motility (data not shown). For IVM (Merck, Darmstadt, Germany) treatment after immunization, mice received 800 µg per kg mouse body weight. For immunization with alum (Thermo Scientific, Bremen, Germany), Mf were added slowly to the adjuvant to a final adjuvant concentration of 25% and then mixed on an automatic shaker at 1,000 rpm for 30 min. After this procedure Mf were morphologically intact, however they were amotile and motility was not reconstituted after 72 h at 37°C and 5% CO_2_, suggesting that the Mf were not viable. Directly before injection, the suspension was intensively vortexed. For the sham injection control mice received alum or PBS. In all experiments, second and third immunization injections were performed two and three weeks after the initial immunization.

### Natural Infection

For challenge infection, infective L3 larvae were transmitted through the bite of the vector mite *Ornithonyssus bacoti* as described [28]. Natural infection was performed one week after the last immunization.

### Mf Monitoring

Peripheral blood was taken from the tail vein and directly transferred into 500 µl Hinkelmann solution (0.5% [wt/vol] eosin Y, 0.5% [wt/vol] phenol (both Merck) and 0.185% [vol/vol] formaldehyde (Sigma-Aldrich) in deionized water). After centrifugation (5 min, 250×g) supernatant was discarded and pellet suspended in 20 µl PBS before counting under the microscope (40×). For monitoring Mf in the pleural space, 20 µl from 1 ml pleural space lavage (see section below) were added to 450 µl Hinkelmann solution and treated as described for peripheral Mf.

### Parasite Recovery

Mice were euthanized with Isofluran (Abbott, Wiesbaden, Germany). Parasites and cells were harvested from the pleural space by lavage with cold PBS. At 15 days p.i., L4 (L3/L4 molting around day eight in BALB/c mice [29]) and at days 70 and 90 p.i. adults (L4/adult molting around day 22 p.i. [29]) were separated from the cells by 15 min sedimentation. For the embryogram each single female worm was transferred into 80 µl PBS, cut into several pieces and embryonic stages squeezed out of the uterus using a 1.5 ml plastic tube and plastic pestle. The embryonic stages were stained by adding 20 µl Hinkelmann solution and 10 µl of this preparation (thus 10% of total uterine content of each analyzed female filariae) were analyzed under the microscope. If present, three female worms from each mouse were investigated. Damaged females, empty females or females with only oocytes were excluded from analysis.

### *Ex vivo* Restimulation

*Ex vivo* stimulations were performed at days 22, 70 or 90 p.i.. Lysis of red blood cells from the pleural space exudate cells was done by 5 min incubation with trisammoniumchloride (Sigma-Aldrich). Cells were washed twice with PBS, filtered through sterile 41 µm gaze (Bueckmann, Moenchengladbach, Germany), and 2.5×10^5^ cells per well in RPMI medium (supplemented with 10% fetal calf serum, 1% L-glutamine, 1% penicillin/streptomycin, 1% non-essential amino acids, 1% sodium bicarbonate, and 1% sodium pyruvate (all PAA)) were stimulated with 5 µg/ml concanavalin A (Sigma-Aldrich) in a 96 well plate (Greiner Bio-One, Frickenhausen, Germany) or the respective worm extracts for 72 h at 37°C and 5% CO_2_. For preparation of *L. sigmodontis* extract, freshly isolated adult worms were rinsed in sterile PBS before being mechanically minced. Insoluble material was removed by centrifugation at 300×g for 10 min and 4°C. Protein concentrations of crude extracts were determined using the Advanced Protein Assay (Cytoskeleton, Denver, CO, USA). All procedures were conducted under sterile conditions. *L. sigmodontis* Mf extract was similarly prepared with sonicated (Bandelin Electronics, Berlin, Germany) freshly isolated Mf.

### Mf-Specific and Cytokine ELISA

Systemic Mf-specific IgG was measured from plasma of mice directly before immunization injections (days −28, −14 and −7) and in weekly intervals after infection (days 0, 7 and 14 p.i.). Blood was taken submandibular from anesthetized (Ketanest, Medistar, Ascheberg, Germany/Rompun, Bayer, Leverkusen, Germany) mice. After centrifugation (5 min at 6,500×g), plasma was taken and stored at −20°C until further usage. Mf-specific Ig of the pleural space were measured from the supernatant of the pleural space lavage at days 22 and 70 p.i.. Polysorb ELISA plates (Nunc, Roskilde, Denmark) were coated overnight at 4°C with 10 µg/ml of Mf crude extract in PBS at pH 9. After blocking 1 h with 1% BSA-PBS (PAA), plates were washed with PBS containing 0.05% Tween 20 (Sigma-Aldrich) and incubated for 2 h at RT with either 50 µl of pleural space lavage or a 1∶10 (IgE) or 1∶1,000 (IgG) dilution of plasma. After another washing step, biotinylated detection antibody (BD Pharmingen, Heidelberg, Germany) was added as recommended by the manufacturer. After a final wash, alkaline phosphatase-conjugated streptavidin (Roche, Grenzach, Germany) was added and tetramethylene benzidine (Carl Roth, Karlsruhe, Germany) was used as substrate. The reaction was stopped by adding 1 M H_2_SO_4_ (Merck) and the absorbance was measured at 450 nm.

IFN-γ (eBioscience, Frankfurt, Germany), IL-5 (BD Pharmingen), IL-13, macrophage inflammatory protein 2 (MIP)-2α, chemokine C-C motif ligand 5 (CCL5), granzyme B, eotaxin-1 and eotaxin-2 (R&D Systems, Wiesbaden, Germany) ELISA of the pleural space lavage and the supernatants of restimulated cells were performed according to manufacturer's instructions.

### Statistics

Statistical analyses were performed with GraphPad Prism 5.0 software (GraphPad Software, La Jolla, CA, USA), using the Student's unpaired t-test for parametric, the Mann Whitney t-test (u-test) for nonparametric data and Welch's correction for data sets with different variances. Variances were tested with the D'Agostino & Pearson omnibus normality test. *P*-values ≤0.05 were considered significant. Microfilaraemia, Ig kinetics and cytokine responses after *ex vivo* restimulation were analyzed with regular 2-way ANOVA and Bonferroni post tests. Data were graphed with means ± standard error of mean (SEM).

## Results

### Subcutaneous Immunization with Mf in Alum Prevents Peripheral Microfilaraemia

**Figure 1:**
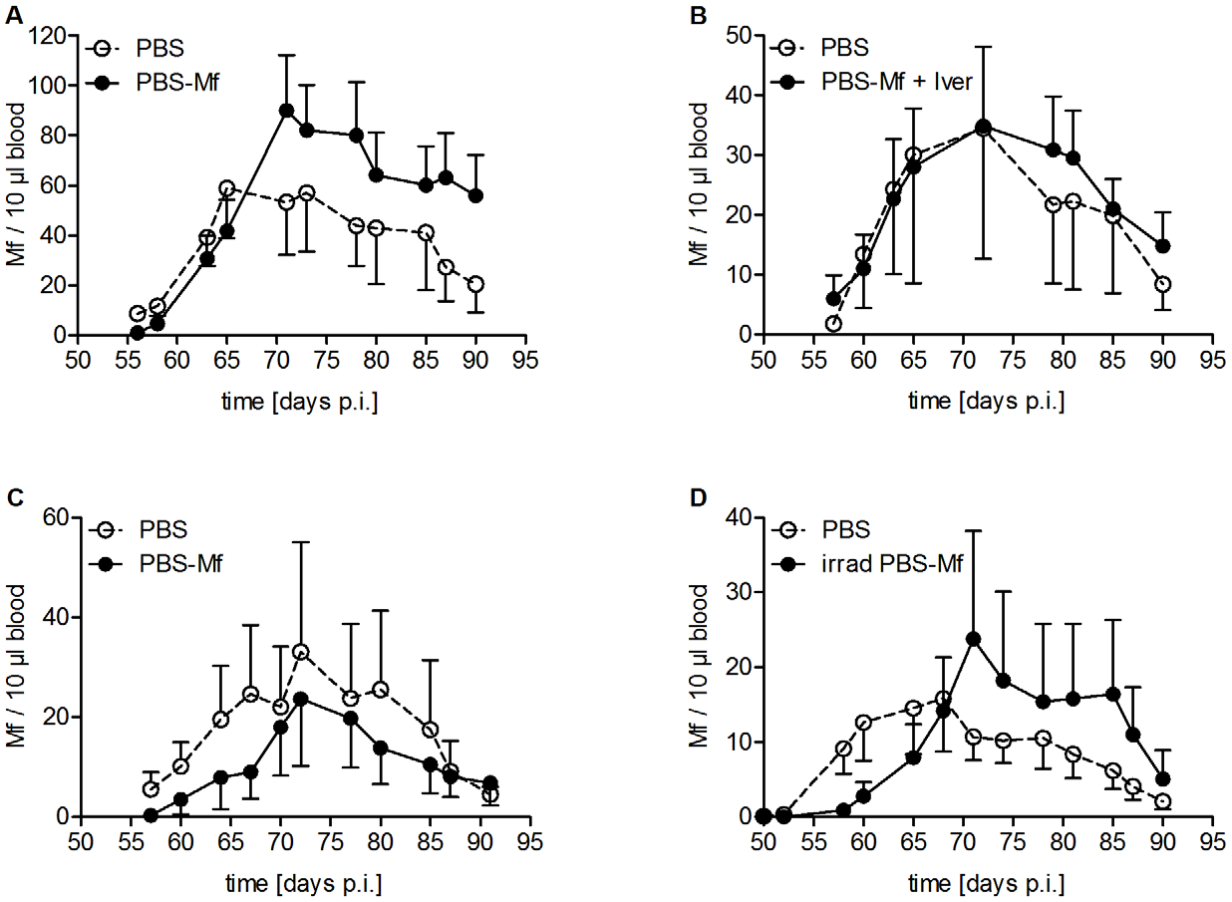
Immunization strategies that failed to protect mice from peripheral microfilaraemia. Mice were immunized with 100,000 Mf either three times i.v. (A, B) or first s.c. followed by an i.p. and i.v. immunization (C, D). All control mice received PBS. *L. sigmodontis* challenge infection was performed one week after the last immunization. (B) After immunization mice were treated i.v. with IVM. (D) Mice were immunized with irradiated (400 Gy) Mf. Microfilaraemia was monitored throughout patency. Data obtained from single experiments with at least six mice per group are shown. Two-way ANOVA (mean ± SEM) was used for statistical analysis including both Mf^−^ and Mf^+^ mice.

Revisiting some of the known immunization protocols in animal models, we immunized mice in various ways (for detailed information see Figure S1). Initially, because Mf are mainly located in the blood of the infected host, we immunized BALB/c mice three times intravenously (i.v.) with 100,000 living Mf. This injection resulted in a transient presence of Mf in the peripheral blood lasting about two weeks (Figure S2A). After challenge infection, natural Mf levels in the peripheral blood were monitored from the onset of peripheral microfilaraemia at day 50 until the end of patency around day 90 p.i.. This immunization neither delayed the onset of natural microfilaraemia nor changed the Mf levels in the peripheral blood after challenge infection compared to control animals at any time point during patency (Figure 1A).

Next, since healthy Mf may modulate immune responses in the immunized host, and in order to enrich the amount of immunogenic material mice were treated after immunization with the microfilaricide IVM, which suppresses the ability of Mf to secrete immunomodulatory proteins [30] and inhibits their neuromuscular control [31]. Accordingly, after IVM injection, Mf disappeared from the peripheral blood within one day after injection (Figure S2B). However, as observed for mice immunized three times with live Mf, microfilaraemia was not reduced in infection-challenged mice (Figure 1B).

According to the successful scheme originally used in cotton rats [24], we then immunized mice first subcutaneously (s.c.), followed by an intraperitoneal (i.p.) immunization two weeks later and an i.v. immunization three weeks after primary immunization. As with the two former schemes, this route of immunization failed to protect mice and this was independent of the usage of either healthy (Figure 1C) or irradiation-attenuated Mf (Figure 1D).

**Figure 2:**
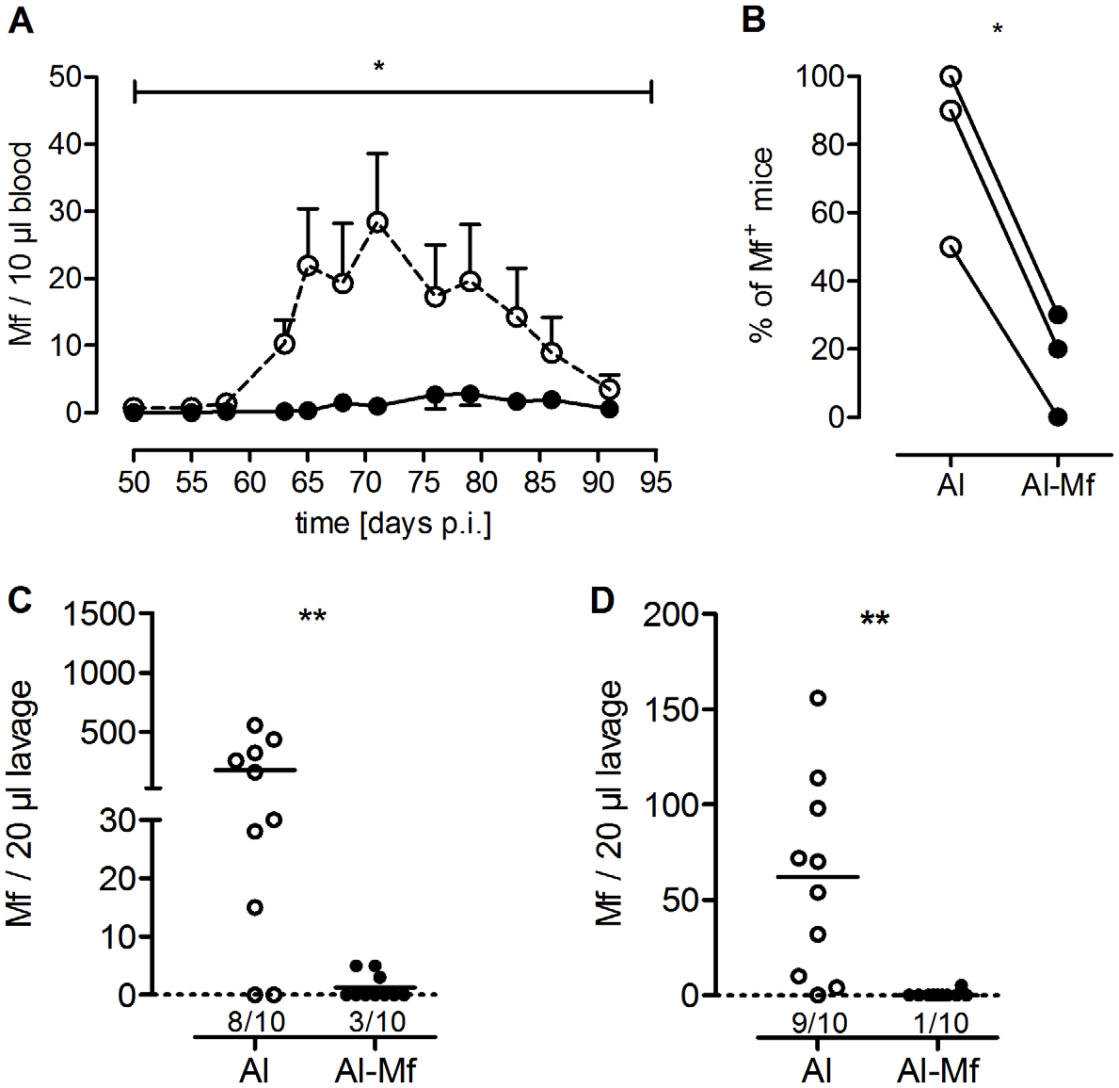
Mice immunized with Mf in alum have reduced numbers of Mf. Mice were immunized three times s.c. with 100,000 Mf in alum. Control mice received alum alone. *L. sigmodontis* infection was performed one week after the last immunization. Microfilaraemia was monitored twice a week throughout patency. (A) Kinetics of Mf load of sham-treated (dashed line) and immunized (black line) mice in the peripheral blood. One representative of three independent experiments with ten mice per group is shown (2-way ANOVA, mean ± SEM), including both Mf^−^ and Mf^+^ mice. For additional experiments see figure S3A, B. (B) Percentage of Mf^+^ mice of three independent experiments was analyzed using Student's t-test. Each mouse with peripheral Mf at any given time point was defined as Mf^+^. (C, D) Mf burden in the pleural space days 70 (C) and 90 (D) p.i.. Graphs show one representative of three (C) and two (D) independent experiments (at least seven mice each group, see also Figure S3C–E) and were analyzed with Welch-corrected t-test. Numbers below the symbols indicate the number of Mf^+^ mice (median, * *P*<0.05, ** *P*<0.005).

Finally, to investigate whether a standard adjuvant is able to establish protective immunity, mice were then immunized three times with 100,000 Mf together with the adjuvant alum. Due to the viscosity of alum this immunization was performed s.c.. Mice immunized with Mf in alum had significantly reduced numbers of circulating Mf after challenge infection compared to control animals throughout patency (*P*<0.05, Figure 2A). Furthermore, the frequency of mice that became microfilaraemic until the end of observation was significantly reduced in the immunized group (*P*<0.05, Figure 2B).

Taken together, effective vaccination of 70–100% was only observed in mice after s.c. immunization with Mf in alum, but not after immunization with Mf alone, irrespective of the administration route, irradiation of Mf or IVM treatment of mice after immunization. Consequently, all further experiments were performed with 100,000 s.c.-administrated Mf in alum.

### Immunization Blocks Embryogenesis of Female Worms

To investigate whether immunization inhibits the ability of females to generate and release Mf or just hinders the Mf migration into the blood, the pleural space lavage was analyzed for the presence of Mf on days 70 and 90 p.i.. Figure 2C and D show, that the number of Mf in the pleural space was significantly reduced after immunization compared to the alum-treated control group, the latter showing a wide range of microfilaraemia that is well described for this model [28]. The few immunized mice that were Mf^+^ at day 70 p.i. had only low Mf levels with a mean of four Mf compared to 226 Mf/20 µl lavage in the alum treated control group (*P*<0.005, Figure 2C). Furthermore, at day 90 p.i. 90% of the immunized mice were free of Mf with only one mouse having five Mf/20 µl lavage. In contrast, 90% of control mice still harbored Mf with a mean of 68 Mf/20 µl lavage (*P*<0.005, Figure 2D). To rule out that alum itself influences the course of infection, we compared mice injected s.c. with either alum or PBS and did not find significant differences in the course of infection (data not shown, 2-way ANOVA of peripheral microfilaraemia *P* = 0.4898, Welch-corrected t-test of pleural space Mf *P* = 0.7377).

**Figure 3:**
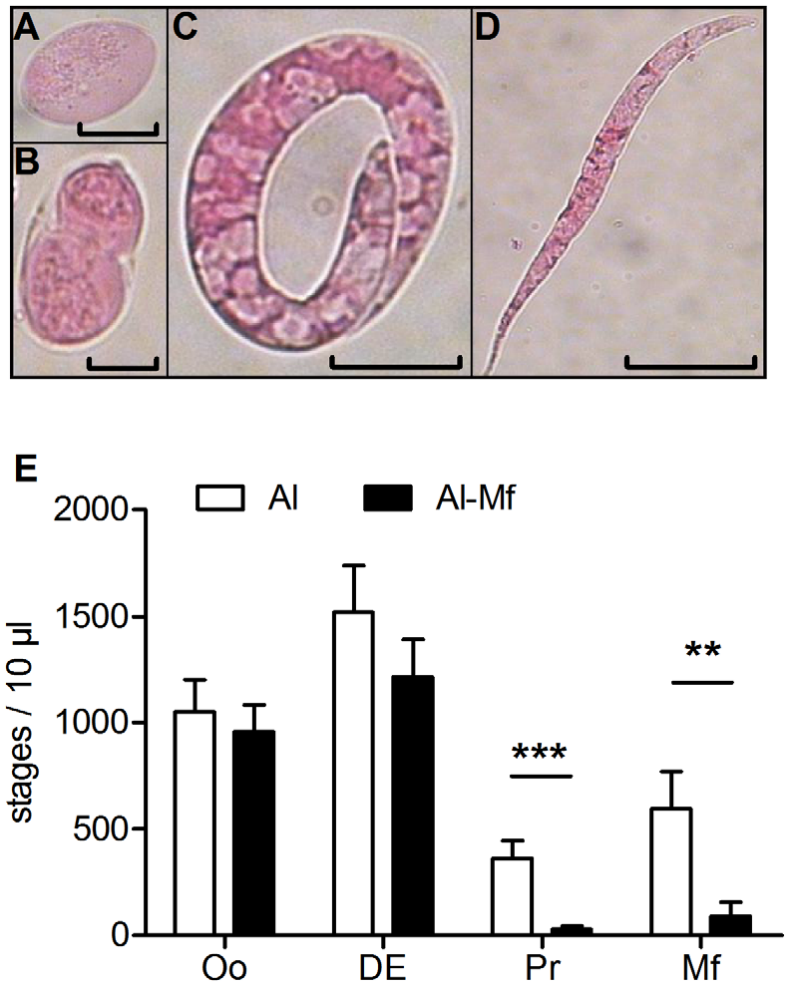
Immunization inhibits embryogenesis in female worms. Mice were immunized three times s.c. with 100,000 Mf in alum. Control mice received alum alone. *L. sigmodontis* challenge infection was performed one week after the last immunization. Seventy days after infection female worms were analyzed for their embryonic stages. Representative pictures of oocyte (A; micron bar 10 µm), divided egg (B; 10 µm), pretzel stage (C; 15 µm) and stretched Mf (D; 30 µm) are shown. (E) Embryogram illustrating the composition of embryonic stages in female worms. If present, three female worms of each mouse were investigated (27 females in the control group, 28 females from the immunized group, additional experiments see Figure S4). Statistical analysis was performed with Mann-Whitney U-test (mean ± SEM, ** *P*<0.01, *** *P*<0.001).

The reduction of Mf levels not only in the blood but also in the pleural space suggested that either Mf were cleared immediately after being released or the Mf output of female worms was reduced. Consequently, the embryogenesis of female worms was analyzed. During the filarial embryogenesis four main developmental stages can be distinguished in the uteri of female worms (Figure 3A–D): oocyte, divided egg (fulfilled first cell division), pretzel and stretched Mf [32], [33]. If present, three female worms from each mouse were investigated. Empty females or females with only oocytes were excluded from analysis. In the embryograms of three independent experiments the percentage of those excluded females was similar between immunized (33, 20 and 27%) and non-immunized mice (16, 18, and 28%), indicating that immunization did not interfere with insemination. At day 70 p.i. we found all stages to be present in the uteri of female worms of control mice, whereas females of immunized mice contained mainly the first two developmental stages (oocyte and divided egg) but rarely pretzel stages (*P*<0.001) and fully developed Mf (*P*<0.01, Figure 3E, see also Figure S4A). To confirm this, any remaining worms were checked for the presence or absence of later stages such as fully stretched Mf and pretzel stages. Only two out of 23 female filariae were positive for later stages in the Mf-vaccinated group (see also Figure S4A; 0/6 worms), whereas in the control group 25 of 31 females contained stretched Mf (see also Figure S4A; 14/26 worms). Inhibition of embryogenesis at day 70 p.i. was exemplarily documented by live video analysis of the uteri of freshly isolated healthy females (video S1, and Video S2). Finally, in an additional experiment inhibition of embryogenesis could already be observed at the beginning of patency (day 56 p.i., see Figure S4B).

Taken together, these data suggest that immunization induces the inhibition of larval development.

### Immunization Reduces Adult Worm Burden

**Figure 4:**
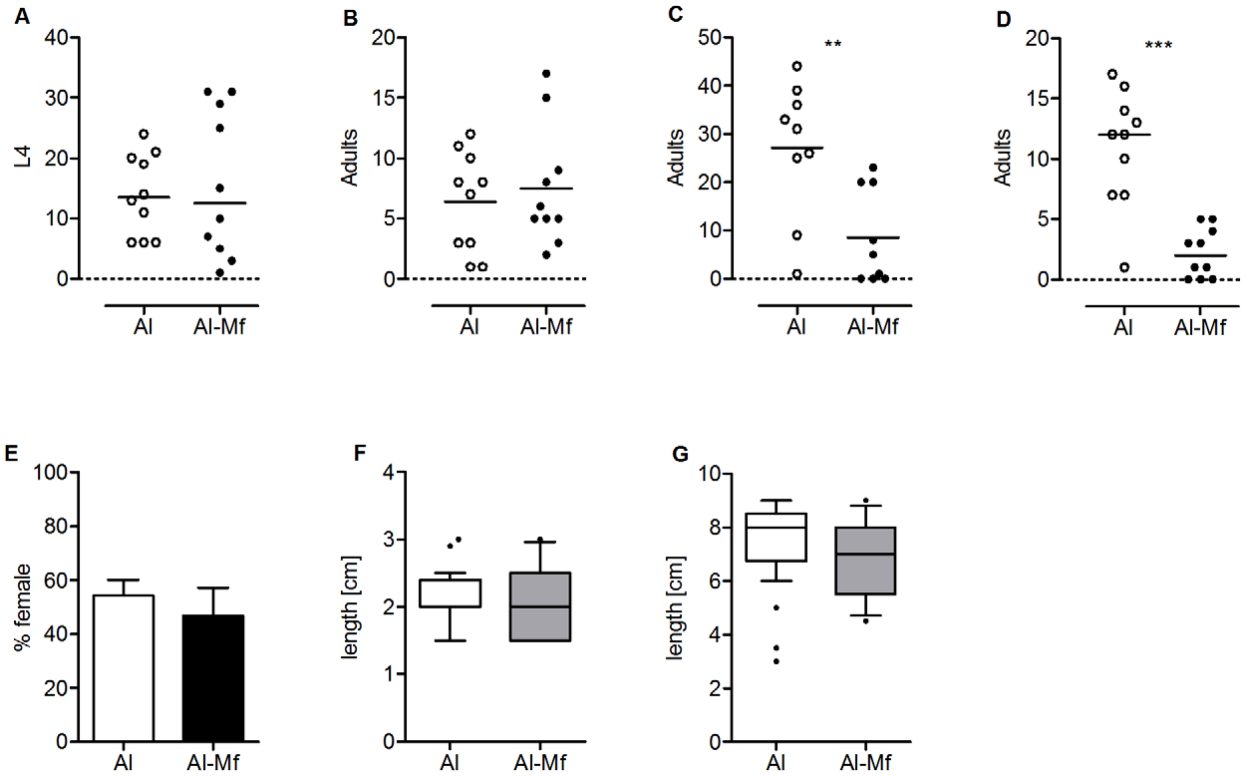
Immunization reduces adult worm burden, but does not affect their development. Mice were immunized three times s.c. with 100,000 Mf in alum. Control mice received alum alone. *L. sigmodontis* challenge infection was performed one week after the last immunization. Numbers of worms on days 15 (A), 56 (B), 70 (C) and 90 (D) p.i. (additional experiments see Figure S5A–C), gender balance (E) (individual experiments see Figure S5D, E), as well as length of males (F) and females (G) at day 90 p.i. (10/90 percentile, outliers are indicated, individual experiments see Figure S5F–I) were analyzed with Student's t-test (** *P*<0.01, *** *P*<0.001).

Cross reactive protection with respect to other developmental stages is known for immunization with *L. sigmodontis* L3. Thus, we asked whether Mf immunization may also affect stages other than the Mf. Analysis of the L4 burden at day 15 p.i. and adult burden at day 56 p.i. showed that immunized mice had similar worm numbers as control animals (Figure 4A, B). However, at day 70 (*P*<0.005, Figure 4C) and 90 p.i. (*P*<0.0001, Figure 4D) immunized mice contained significantly fewer adult worms and this reduction was associated with decreased numbers of both males and females, as the gender balance was similar in immunized and control mice (Figure 4E). Male and female worms did not differ in length to the corresponding worms of control mice (Figure 4F, G).

Taken together, our data show that immunization with 100,000 Mf in alum not only inhibited microfilaraemia, but also reduced adult worm burden at later time points.

### Humoral and Cellular Immune Responses Induced by Immunization

**Figure 5:**
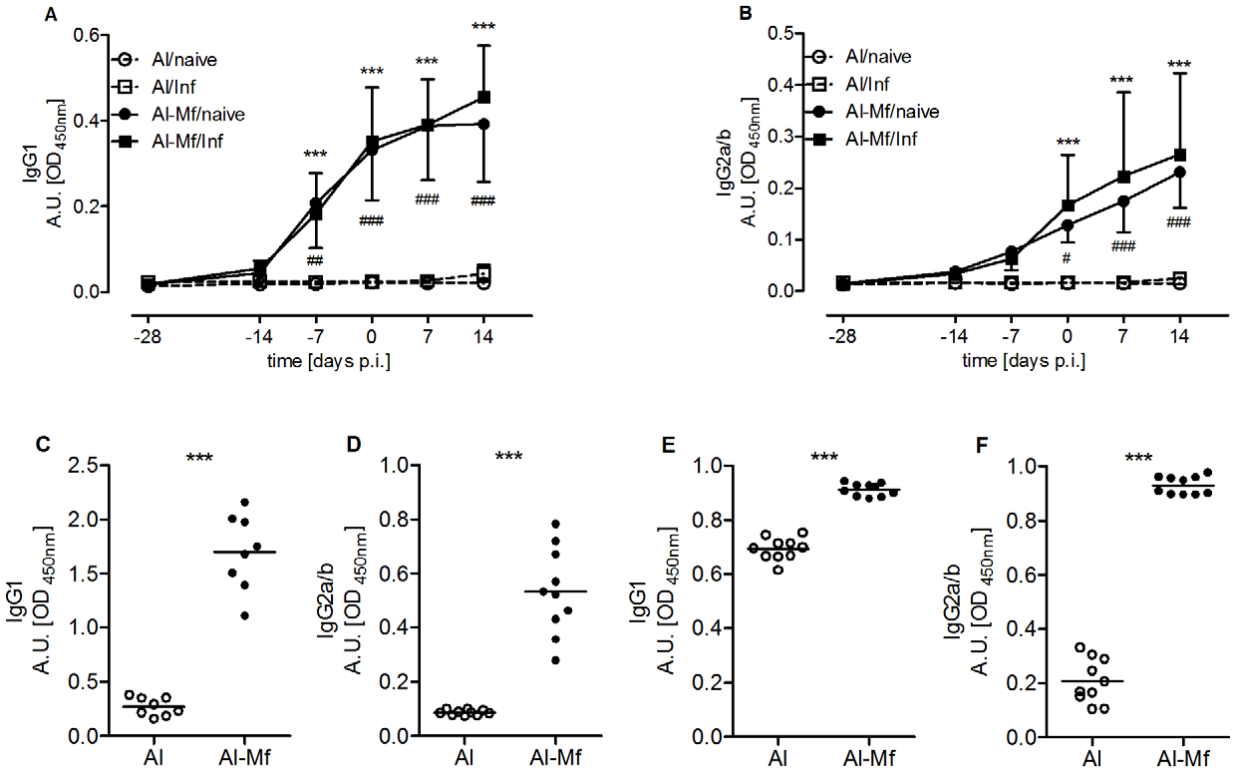
Immunization induces Mf-specific IgG1 and IgG2. Mice were immunized three times s.c. with 100,000 Mf in alum (Al-Mf/naïve, Al-Mf/Inf). Control mice received alum alone (Al/naïve, Al/Inf). *L. sigmodontis* challenge infection was performed one week after the last immunization (Al/Inf, Al-Mf/Inf) or left uninfected (Al/naïve, Al-Mf/naïve). Plasma levels of Mf-specific IgG1 (A) and IgG2a/b (B) were measured. Two-way ANOVA was used for statistical analysis, day 0 indicates day of challenge infection. Asterisks indicate significant differences between the immunized and infected, and the corresponding control group (*** *P*<0.001) and pound signs between the immunized but uninfected, and the corresponding control group (^#^ *P*<0.05, ^##^ *P*<0.01, ^###^ *P*<0.001). (C–F) Pleural space lavage was analyzed for specific IgG1 and IgG2a/b on days 22 (C, D) and 70 p.i. (E, F). Data analyzed with Welch-corrected t-test (mean, *** *P*<0.001). Graphs show representatives of three independent experiments with eight to ten mice each group (additional experiments see Figure S6A, B, E–J).

To investigate whether immunization-induced Mf-specific Ig responses were associated with protection, Mf-specific IgE, IgG1 and IgG2 levels were measured in the plasma and pleural space lavage at different time points throughout immunization and infection. As Figure 5A and B illustrate, the immunization induced an Mf-specific humoral response and both IgG1 and IgG2 antibodies were elevated in the blood. The most prominent increase was observed after the boost immunizations, as indicated by the levels seven days before the challenge infection. A comparison of both immunized groups (infected vs. uninfected) revealed that these humoral responses were not further enhanced by the infection itself. The same picture was found at the site of infection with Mf-specific IgG1 and IgG2 levels being significantly elevated in immunized mice compared to controls on day 22 (*P*<0.001, Figure 5C, D) as well as on day 70 p.i. (*P*<0.001, Figure 5E, F). Albeit the differences in IgG1 levels remained significantly higher in immunized mice at day 70 p.i., the IgG1 levels of infected but non-immunized mice increased on days 28 and 42 (Figure S6C, D) compared to day 22 p.i. (Figure S6A, B). This however indicates a Th2 shift induced by the parasite itself and is well-known for primary infected BALB/c mice [34], [35].

The amount of Mf-specific IgE was not increased at day 22 p.i. in the pleural space of immunized mice (mean OD of 0.047) compared to non-immunized mice (mean OD of 0.0892). Later during infection, Mf-specific IgE was elevated in the blood of immunized mice with a mean OD of 0.121 (day 28 p.i.) and 0.187 (day 42 p.i.) in immunized mice, and a mean OD of 0.043 and 0.050 in control mice, respectively. However, these levels of Mf-specific IgE clearly did not reach the IgE levels of chronically infected mice (OD on day 100 p.i. 1.683; single experiment, data not shown), suggesting that immunization *per se* does not lead to a strong IgE induction.

**Figure 6:**
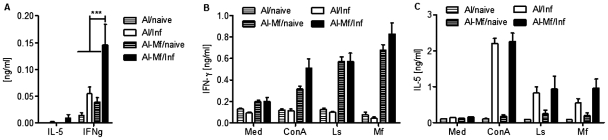
Immunization enhances IFN-γ responses. (A) At day 22 p.i. the pleural lavage was analyzed for IL-5 and IFN-γ. Combined data of three independent experiments with five mice each group are shown. (B, C) At day 22 p.i. cells from the site of infection were restimulated for 72 h with 5 µg/ml Concanavalin A (ConA), 100 µg/ml complete adult (Ls) or microfilarial (Mf) crude extract of *L. sigmodontis* and IFN-γ (B) and IL-5 (C) secretion were measured (mean ± SEM). Representative data of two independent experiments with five mice each group. Analysis was done using the 2-way ANOVA, for significances see text.

To classify cellular responses, we analyzed major cell populations in the pleural space lavage by flow cytometry. However, no consistent differences were observed (Table S1). We also measured various cytokines in the pleural space at day 22 p.i.. Since eosinophils are known effector cells in helminth infections [36], we measured molecules involved in eosinophil recruitment or activity, i.e IL-13, MIP-2α, CCL5, granzyme B, eotaxin-1 and eotaxin-2. Results from three independent experiments did not reveal any significant differences between immunized mice and control animals (data not shown). However, analysis of hallmark cytokines IL-5 and IFN-γ of type 1 and 2 immunity showed that immunized mice had significant more IFN-γ in the pleural space of the thoracic cavity (*P*<0.001), whereas level of IL-5 were low irrespective of the immunization (Figure 6A). A similar picture was observed when cells recovered from the pleural space were restimulated with worm extracts (Figure 6B, C). Strikingly, 22 days p.i., a time point when parasites are already present in the pleural space, only cells of immunized mice secreted IFN-γ regardless of whether they were infected or not. This effect was seen after specific restimulation with crude extract of adult worms and Mf, as well as with nonspecific stimulation by concanavalin A (*P*<0.001, Figure 6B). Although less pronounced, enhanced IFN-γ responses after restimulation were also present throughout patency (Figure S7A, B). Different to IFN-γ, the IL-5 responses were dependent on the infection itself, as only cells from infected mice secreted IL-5 after restimulation irrespective of immunization (Figure 6C, Figure S7C, D).

## Discussion

Current public health control of human filarial infections relies on chemotherapy provided by MDA programs. Antifilarial drug therapy has to be implemented for years with high coverage, incurring high logistical costs and the emergence of drug resistance is a potential threat [12]. Thus, a vaccine that results in the reduction of parasite burden would complement the MDA efforts, as suggested for other neglected tropical diseases [37] and that would be complementary step towards elimination of the diseases.

The present study describes a successful immunization protocol against *L. sigmodontis* Mf in the murine model of filariasis, which additionally resulted in a reduced adult worm burden. Subcutaneous immunization with Mf in alum prevented the onset of microfilaraemia after challenge infection in the majority of mice. Reduced Mf loads were observed in the peripheral blood and at the site of infection in conjunction with intrauterine inhibition of embryogenesis. Protection was further associated with systemic and local Mf-specific IgG and IFN-γ secretion of pleural space exudate cells.

In mouse models the adjuvant alum is often administered i.p. and this route has been referred to establish “systemic” responses in contrast to the “local” s.c. route [38]. Interestingly, we found neither the systemic i.v. route nor the combination of the local s.c. and both systemic i.p. and i.v. routes to be able to reduce microfilaraemia (Figure 1). As systemic immunizations have been reported to induce tolerance rather than immunity [39], out of the protocols tested in this study, only the s.c. immunization was able to immunize mice successfully against *L. sigmodontis* Mf. The s.c. immunization would also be a route, which is best applicable to humans.

It is known that immunization with irradiated L3 stages reduces the recovery rate of larvae in the pleural space [40]. However, immunization with Mf did not render mice less susceptible for infection *per se*, as the worm burden did not differ on day 15 p.i.. The short time between immunization and challenge infection might explain the absence of enhanced immunity against incoming L3. The observation that the adult worm burden remained similar in both groups until the onset of patency at day 56 p.i., may suggest that the accessibility of Mf in the pleural space of the thoracic cavity could be a critical step in initiating the responses that affect the adult filariae. However, only one experiment was performed on day 56 and a more detailed analysis of the efficacy on adult worm burden is needed to identify the time point when adulticidal immune responses are effective. Interestingly, at later time points, namely on days 70 and 90 p.i., we could observe a reduced worm burden in immunized mice compared to the controls. This reduction was seen with both male and female worms, suggesting effector mechanisms not only acting against intrauterine Mf in female worms, but against target structures of adult worms. In line with this, different developmental stages of filariae share many molecular structures [41] and cross-reactive immunization effects are documented for filarial immunizations [19], [42]. Importantly, it is unlikely that the lower worm burden observed on days 70 and 90 p.i. is the reason for reduced microfilaraemia in immunized mice, because all immunized mice contained male and female worms and it has been shown that even a few fecund females can establish peripheral microfilaraemia [43].

Inhibition of embryogenesis in immunized mice was further indicated by the reduced number of Mf in the pleural space and, more importantly, the presence of embryonic stages that had not developed beyond several divisions of the fertilized oocytes. This is in contrast to Wenk and colleagues who found fully developed Mf in the uterus of female worms and in the pleural space of infected and Mf-immunized cotton rats [23]. The differences between both animal models in the mode of Mf reduction may be due to different susceptibilities of the hosts to *L. sigmodontis*. Both, BALB/c mouse and the cotton rat develop patent infection, but BALB/c mice clear the infection after 3–4 months, whereas the cotton rat is the natural host and can harbor filarial parasites for years [29]. This is the general downside of using laboratory mice for *L. sigmodontis* infection rather than the natural host; however the advantage is that cytokine responses can be measured and associated with protection, and for future studies cytokine deficient mice may answer further questions about the essentiality of key cytokines for vaccination success.

In our experiments, immunization induced Mf-specific IgG1 and IgG2 antibodies, present throughout the whole infection including patency, were associated with protection. Because protective responses induced by immunization often rely on protective antibodies [44] and the importance of B cells in promoting immune responses against filarial Mf is well documented for the murine model of filariasis [45], [46], we analyzed the frequency and total number of pleural space B1 and B2 B cells by flow cytometry, but found no consistent differences between immunized and non-immunized mice (Suppl. Table 1), suggesting differences in B cell activation rather than in total B cell numbers. Interestingly, despite a strong antibody production female worms of immunized mice appeared morphologically as intact as those in non-immunized control mice. Furthermore we did not find any host cells within the female worms. These two findings suggest a blockade of embryonic development, rather than a cell-dependent destruction of the embryonic stages. In our experiments, Mf-specific IgGs may have entered the female worm uterus and bound to the developing early developmental stages, thereby hindering their further growth in an Ig-dependent, but cell-independent manner. Indeed, it is known that filarial-infected humans produce filarial-specific Ig that is able to bind early intrauterine filarial stages, as shown by using sera of chronic LF patients against isolated intrauterine Mf stages from the filariae *Setaria digitata* [47]. Another possibility for cell-independent, Ig-mediated responses is the activation of the complement cascade resulting in the formation of the membrane attack complex (MAC), due to insertion of complement proteins into a phospholipid bilayer [48]. Although there is as yet no evidence for MAC formation in the sheath of adult nematodes, earlier developmental stages may be more sensitive to MAC formation. The next step to clarify the role of antibodies in the establishment of immunization-induced protection would be the verification of embryonic stage-bound Mf-specific Ig, e.g. by immunohistochemistry. Furthermore, experiments with mice having a defect in immunoglobulin production would give important insights into the relevance of Ig for impairment of embryogenesis. Also, a possible IgE reaction to the immunization has to be elucidated. We did not observe a strong IgE response after immunization and infection in the blood nor at the site of infection. However, future experiments should clarify how chronically infected mice that already have Mf-specific IgE respond to immunization. In humans, a vaccination may be favourable one month after IVM treatment, when individuals have no skin or blood Mf, as the risk of urticaria due to immune attack on remaining Mf would be at its minimum.

IFN-γ and IL-5 are well known players in innate, adaptive and vaccine-induced immunity against helminths [49], with the adaptive type 2 response referred to as “typical” for helminth infections [50]. We found that immunization was associated with strong IFN-γ responses, mirrored by increased levels in the pleural space and after restimulation of pleural exudate cells. The importance of IFN-γ production in immune responses against Mf in permissive BALB/c mice is underlined by several findings. IFN-γ^−/−^ mice have increased numbers of circulating *L. sigmodontis* Mf compared to the wild type littermates [51]. In addition it has been shown that IFN-γ RNA levels of restimulated splenocytes obtained from *L. sigmodontis*-infected BALB/c mice are strongly increased within days after the beginning of patency [52]. These observations may reflect the moderate increase in IFN-γ production also in non-immunized mice upon natural infection and the less pronounced but still significant differences in IFN-γ between immunized and non-immunized mice during patency (Figure S7A, B). Furthermore, it is known that injected *B. malayi* Mf, but not implanted adult stages induce IFN-γ and Th-1-assiociated IgG2a in BALB/c mice [53]. IFN-γ is an inducer of IgG2a [54] therefore it is most likely that in our experiments, Mf-induced IFN-γ has promoted the secretion of IgG2. Importantly, induction of IFN-γ is not in conflict with the use of the adjuvant alum, which is generally referred as Th2-promoting, because recent findings have shown that alum also can influence proliferation and IFN-γ production of CD8^+^ T cells [55]. Furthermore, Toll-like receptor agonists have been found to be able to bias alum towards a mixed Th1/Th2 response [56]. *L. sigmodontis*, like many other filariae contains endosymbiotic *Wolbachia* bacteria that are recognized by Toll-like receptors [57].

Although IL-5 responses did not differ between immunized and control mice, this cytokine may also play a role for the overall effect of immunization in the infected mice. It may even be possible that the effect of immunization, although predominated by IFN-γ, may be dependent on at least baseline levels of IL-5, since this cytokine has been shown to be important for both adult worm and Mf containment in *L. sigmodontis* infection in our earlier reports [58], [59]. Future immunization experiments with BALB/c mice defective for IFN-γ or IL-5 responses will shed more light on the importance of both key cytokines for the inhibition of embryogenesis.

Taken together, the immunization scheme presented in this study demonstrates the feasibility of an immunization that is directed against the Mf stage, leading to protection against peripheral microfilaraemia with an efficacy of up to 100%. The IFN-γ that has been induced by the immunization suggests a shift towards a Th1-like milieu in the host that may furthermore promote direct or indirect responses against the Mf during patency, possibly through IFN-γ-promoted IgG2a. It is known for human LF that there is a threshold for Mf density in the peripheral blood to achieve transmission and a high number of infective bites is needed to produce a patent infection [60]. We hypothesize that the reduction of circulating peripheral Mf at the level we observed might prevent transmission.

The study presented here contributes to the understanding of the immune mechanisms needed to develop a vaccine against filarial parasites. Whereas the use of Mf recovered from infected humans would be costly and the number of Mf limited, even disregarding the potential transmission of other infections, our data may serve for a better understanding of the nature of protective Mf vaccination. Future assessments should address the characterization of microfilarial molecular subunits that account for this protection, as the growing fields of helminth genomics [61] and systems biology [62] may predict such potential Mf-related vaccine candidates. Administration of only a subunit vaccine may also avoid vaccination with tolerogenic molecules contained within the Mf and lead to better efficacy of protection.

## Supporting Information

Figure S1 **Immunization schemes.** Figure shows schedule of immunization, challenge and analysis. Table shows detailed information of all immunization experiments mentioned in the text.
(TIF)

Figure S2 **Kinetic of i.v.-injected Mf in the peripheral blood.** 100,000 Mf were injected i.v. into the tail vein and Mf in the peripheral blood was monitored daily. (A) Representative data of two independent injections with seven mice per group (mean ± SEM). (B) One hour after injection mice were treated with IVM at 800 µg per kg body weight. One representative of two independent injections with eight mice per group is shown (mean ± SEM).
(TIF)

Figure S3 **Additional experiments showing reduced Mf load.** Mice were immunized three times s.c. with 100,000 Mf in alum. Control mice received alum alone. *L. sigmodontis* infection was performed one week after the last immunization. (A, B) Kinetics of Mf load of control (dashed line) and immunized (black line) mice in the peripheral blood of two additional experiments with ten mice per group each experiment (2-way ANOVA, mean ± SEM), including Mf^−^ and Mf^+^ mice. (C–E) Mf burden in the pleural space at days 70 (C, D) and day 90 (E) p.i., analyzed with Welch-corrected t-test (mean, * *P*<0.05). Numbers below the symbols indicate number of Mf^+^ mice in the shown experiment.
(TIF)

Figure S4 **Additional experiments illustrating inhibited embryogenesis in female worms of immunized mice.** Mice were immunized three times s.c. with 100,000 Mf in alum. Control mice received alum alone. *L. sigmodontis* challenge infection was performed one week after the last immunization. Seventy (A) and 56 days (B) after infection female worms were analyzed for their embryonic stages. Analysis was performed with Mann-Whitney u-test (mean ± SEM, * *P*<0.05, ** *P*<0.01, *** *P*<0.001).
(TIF)

Figure S5 **Additional data illustrating that immunization reduces adult worm burden, but does not affect their development.** Mice were immunized three times s.c. with 100,000 Mf in alum. Control mice received alum alone. *L. sigmodontis* challenge infection was performed one week after the last immunization. Numbers of worms at days 70 (A) and 90 p.i. (B, C), gender balance of worms (D, E) and length of males (F, G) and females (H, I) at day 90 p.i. (10/90 percentile, outliers are indicated) were analyzed with Student's t-test (mean ± SEM, * *P*<0.05, *** *P*<0.001).
(TIF)

Figure S6 **Additional data confirming enhanced Mf-specific IgG1 and IgG2.** Mice were immunized three times s.c. with 100,000 Mf in alum (Al-Mf/naïve, Al-Mf/Inf). Control mice received alum alone (Al/naïve, Al/Inf). *L. sigmodontis* challenge infection was performed one week after the last immunization (Al/Inf, Al-Mf/Inf) or left uninfected (Al/naïve, Al-Mf/naïve). Plasma levels of Mf-specific IgG1 (A) and IgG2a/b (B) were measured. Asterisks indicate significant differences between the immunized and infected, and the corresponding control group (* *P*<0.05, ** *P*<0.01, *** *P*<0.001) and pound signs between the immunized but not infected, and the corresponding control group (^#^ *P*<0.05, ^##^ *P*<0.01, ^###^ *P*<0.001). (C, D) Plasma Mf-specific IgG1 and IgG2 at days 28 and 42 p.i. measured in a single experiment and analyzed with Student's t-test (mean ± SEM, *** *P*<0.001). (E–J) Pleural space lavage was analyzed for specific IgG1 and IgG2a/b at days 22 (E–H) and 70 p.i. (I, J). Data were analyzed with the Welch-corrected t-test (mean, ** *P*<0.01, *** *P*<0.001).
(TIF)

Figure S7 **IFN-γ and IL-5 responses of pleural space exudate cells.** Cells from the site of infection were restimulated 72 h with 5 µg/ml Concanavalin A (ConA) or 100 µg/ml complete adult crude extract of *L. sigmodontis* (Ls) at day 70 (A, C) or day 90 p.i. (B, D). Combined data from two independent experiments are shown. Analysis was done with 2-way ANOVA (mean ± SEM, * *P*<0.05).
(TIF)

Table S1 **Flow cytometric analysis of pleural space exudate cells 15, 22, 70 and 90 days p.i..** Mice were immunized three times s.c. with 100,000 Mf in alum. Control mice received alum alone. *L. sigmodontis* challenge infection was performed one week after last immunization. Percentages (A) and absolute numbers (B) for dendritic cells (DC), macrophages (MO), B-cells (BC) and B2 B-cells (B2 BC), T-cells (TC) and eosinophils (EO) are shown. Staining was performed according to standard protocols with fluorochrome-conjugated antibodies to the surface markers F4/80, SiglecF, CD3ε, CD11c, CD19, and CD23, used as recommended by the manufacturers (eBioscence, BD Pharmingen). “-” indicates that no data are available for that time point.
(XLS)

Video S1 **Live** ***in-uteri*** **analysis of larval development in control mice.** One representative adult female worm isolated from the pleural space of an alum-treated control mouse was placed on a microscope slide and filmed with the microscope analysis software Diskus 4.6 (Hilger, Koenigswinter, Germany). Living and moving Mf in the uterus can be seen.
(MOV)

Video S2 **Live** ***in-uteri*** **analysis of larval development in immunized mice.** One representative adult female worm isolated from the pleural space of an Mf-alum immunized mouse was placed on a microscope slide and filmed with the microscope analysis software Diskus 4.6 (Hilger, Koenigswinter, Germany). Divided eggs stages in the uterus can be seen (indicated by the arrow).
(MOV)
