# Supplementary figures and images for: Immunization with L. sigmodontis Microfilariae Reduces Peripheral Microfilaraemia after Challenge Infection by Inhibition of Filarial Embryogenesis
Source: PLoS Negl Trop Dis. 2012 Mar 6;6(3):e1558. doi: 10.1371/journal.pntd.0001558 (PMC3295809; doi:10.1371/journal.pntd.0001558)

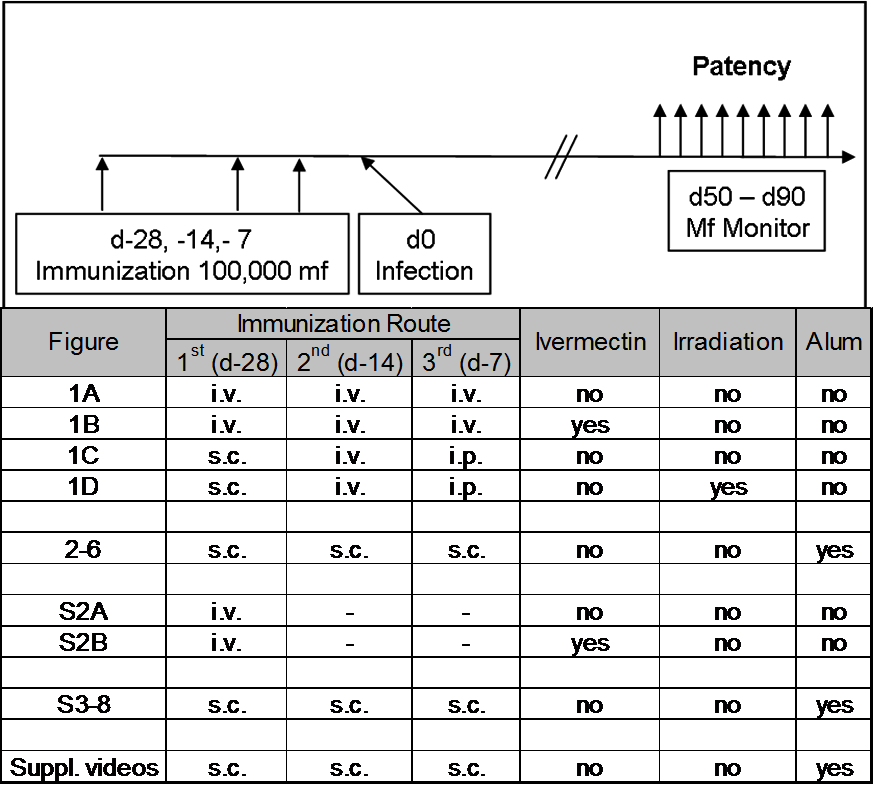

Supplement: Figure S1 — Immunization schemes. Figure shows schedule of immunization, challenge and analysis. Table shows detailed information of all immunization experiments mentioned in the text. (TIF) [file pntd.0001558.s001.tif]
